# Transcatheter aortic valve-in-valve implantation of a CoreValve in a JenaValve prosthesis: a case report

**DOI:** 10.1186/s13256-017-1409-x

**Published:** 2017-09-10

**Authors:** Shahram Lotfi, Michael Becker, Ajay Moza, Rüdiger Autschbach, Nikolaus Marx, Jörg Schröder

**Affiliations:** 1 Department of Thoracic and Cardiovascular Surgery, RWTH University Aachen, Pauwelsstraße 30, 52074 Aachen, Germany; 2 Department of Cardiology (Internal Medicine, Clinic I), RWTH University Aachen, Aachen, Germany

**Keywords:** TAVI, Valve-in-valve, Implantation, Severe aortic stenosis

## Abstract

**Background:**

Transcatheter aortic valve implantation has become an accepted treatment modality for inoperable or high-risk surgical patients with symptomatic severe aortic stenosis.

**Case presentation:**

We report the case of a 70-year-old white man who was treated for severe symptomatic aortic regurgitation using transcatheter aortic valve implantation from the apical approach. Because of recurrent cardiac decompensation 4 weeks after implantation he underwent the implantation of a left ventricular assist device system. A year later echocardiography showed a severe transvalvular central insufficiency. Our heart team decided to choose a valve-in-valve approach while reducing the flow rate of left ventricular assist device to minimum and pacing with a frequency of 140 beats/minute. There was an excellent result and our patient is doing well with no relevant insufficiency of the aortic valve at 12-month follow-up.

**Conclusion:**

This is the first report about a successful treatment of a stenotic JenaValve using a CoreValve Evolut R; the use of a CoreValve Evolut R prosthesis may be an optimal option for valve-in-valve procedures.

## Background

Transcatheter aortic valve implantation (TAVI) has become an accepted treatment modality for inoperable or high-risk surgical patients with symptomatic severe aortic stenosis [1]. Implantation of a second percutaneous heart valve of the same type in a case of severe paravalvular leakage after placement of a first valve prosthesis has been described [2]. Similarly, implantation of a percutaneous heart valve of the same type has been performed for treatment of a degenerated percutaneous heart valve [3]. For the first time this is a report about treatment of an insufficient percutaneous heart valve by a second different type of percutaneous heart valve namely an implantation of a CoreValve Evolut R into a JenaValve. In addition, this case is exciting because this implantation was performed while running a left ventricular assist device (LVAD) system. This case report includes a report of the successful implantation and advice on the specific “landing zone.”

## Case presentation

A 70-year-old white man was treated for severe symptomatic aortic regurgitation due to healed endocarditis using TAVI from the apical approach. TAVI was performed at that time because he was considered a high-risk surgical patient due to secondary pulmonary hypertension, severely impaired left ventricular function with a left ventricular ejection fraction (LVEF) of 20%, chronic renal failure, and a logistic EuroSCORE I of 24.36%. At the time he was treated by diuretics (torasemide 20 mg once a day), an angiotensin-converting enzyme (ACE) inhibitor (ramipril 5 mg once a day), a ß-blocker (bisoprolol 2.5 mg twice a day), and an aldosterone antagonist (12.5 mg once a day). On admission he had cardiac decompensation and resulting dyspnea (temperature 36.7 °C, pulse 99/minute, blood pressure 109/48 mmHg) but his emotional status and neurological constitution were good. The laboratory results were unremarkable except for: a mild increase in liver enzymes, aspartate aminotransferase (AST) 59 U/l and alanine aminotransferase (ALT) 67 U/l; a known chronic renal insufficiency (creatinine 2.1 mg/dl); and a mild decrease in hemoglobin (Hb) 10.7 g/dl. No urine analysis was done. Due to normal C-reactive protein and normal count of leukocytes no microbiological examination was performed. After interdisciplinary discussion of the case (including a normal coronary angiography that was performed a few days before) and cardiac recompensation, he was initially treated with an implantation of a JenaValve 27 mm self-expandable valve. Despite a good result after implantation with the JenaValve and minimal transvalvular central insufficiency, he presented recurrent cardiac decompensation due to his severely impaired LVEF. His case was discussed again at an interdisciplinary meeting: 4 weeks after TAVI he underwent the implantation of a LVAD system (Thoratec® HeartMate II). His postoperative course was uneventful. He remained asymptomatic for 1 year until the LVAD system showed recurrent significant high flow alarms. Echocardiography examinations during this year showed a continuous increase in transvalvular central insufficiency to the level of a severe regurgitation without any sign for structural alteration of the leaflets of the JenaValve prosthesis. Treatment options were discussed and a new TAVI as valve-in-valve was decided.

**Fig. 1:**
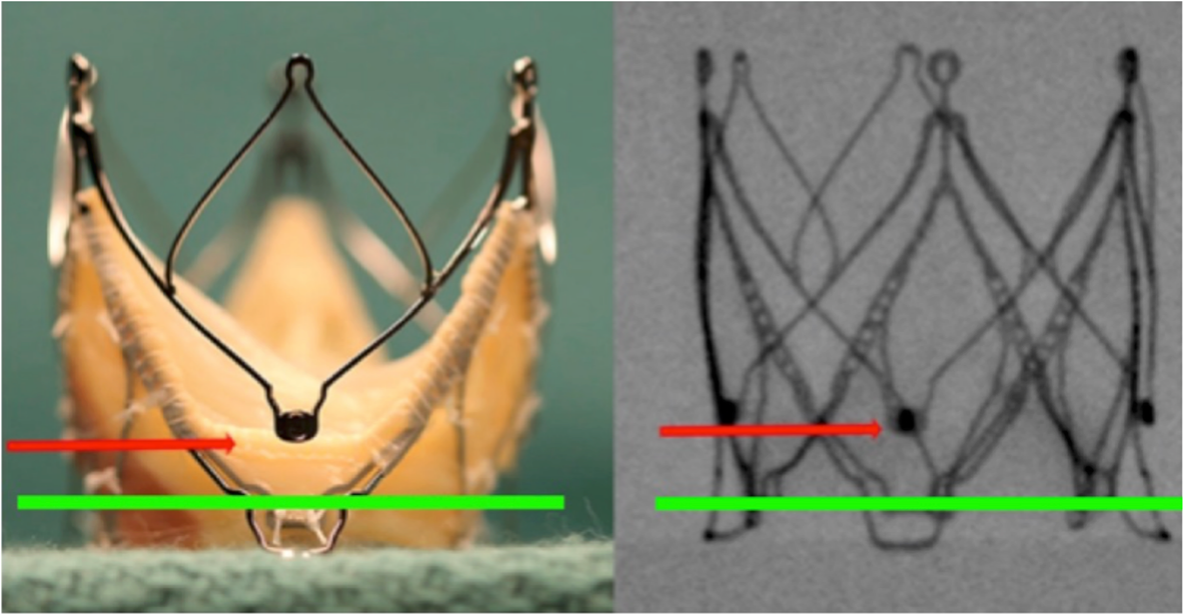
Planning of intervention using the app “Valve in valve” by Dr Vinayak Bapat. The *red marker* shows the “cusp feelers”, the *green line* shows the intended “landing zone” of the new valve

**Fig. 2:**
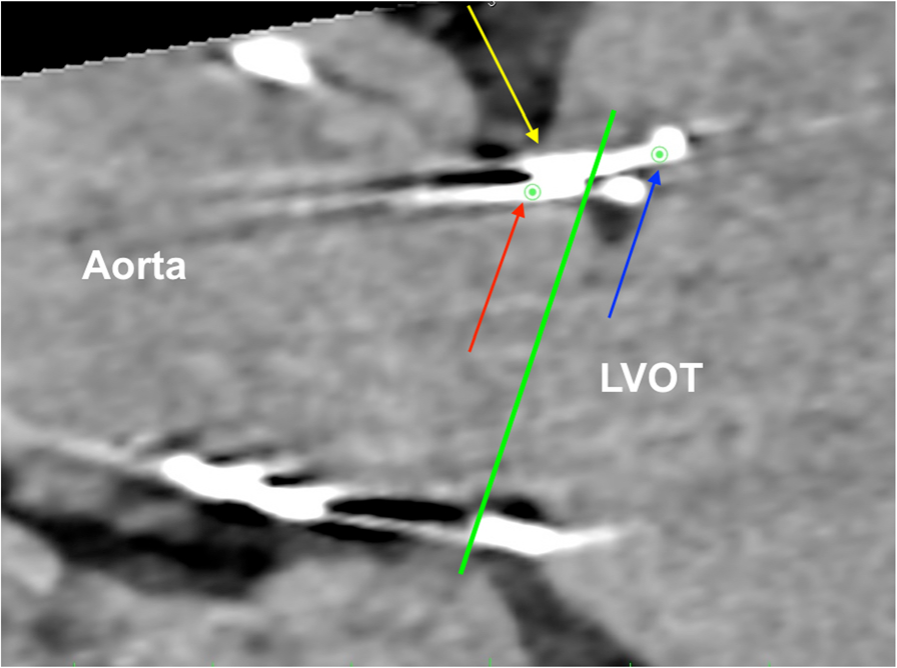
Additional planning of intervention using cardiac computed tomography: given is the analysis for left coronary cusp (analyses of right and non-coronary cusp are not shown). *Red arrow*, “feeler” of JenaValve; *blue arrow*, lowest point of JenaValve; *yellow arrow*, aortic annulus in at deepest point in left coronary cusp; *green line*, intended level for positioning of the lowest point of CoreValve Evolut R prosthesis determined by our Transcatheter aortic valve implantation-Team (not above the original aortic annulus and not deeper than lowest point of JenaValve avoiding possible interference with mitral valve). *LVOT* left ventricular outflow tract

**Fig. 3:**
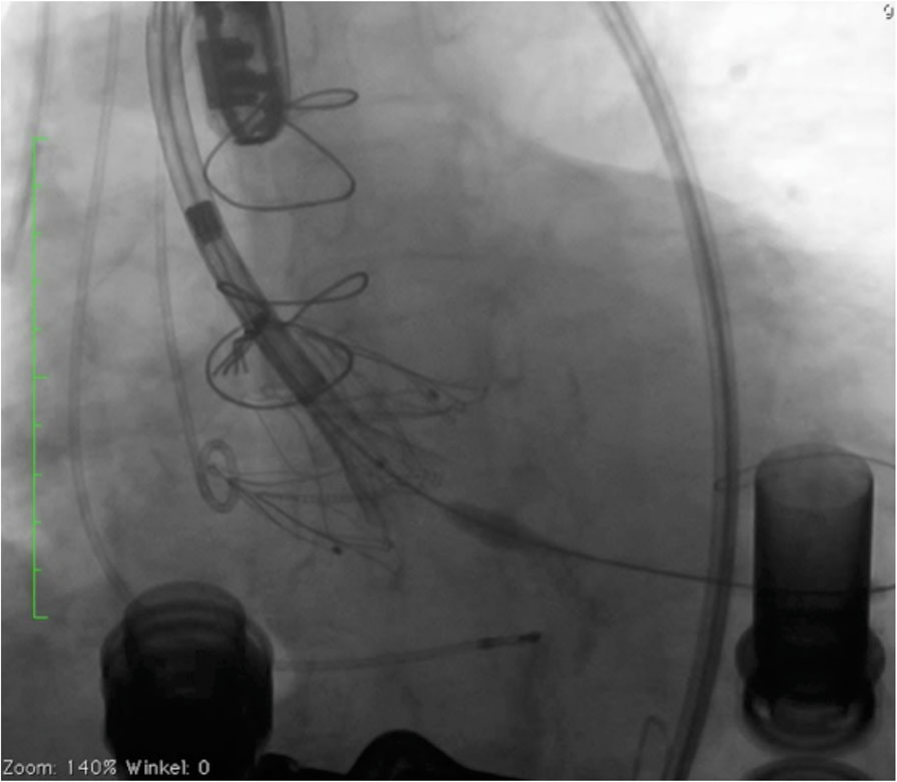
Fluoroscopy during implantation of the CoreValve prosthesis within the JenaValve prosthesis demonstrating the applied position with the distal end being positioned between the “cusp feelers” and the distal end of the JenaValve

**Fig. 4:**
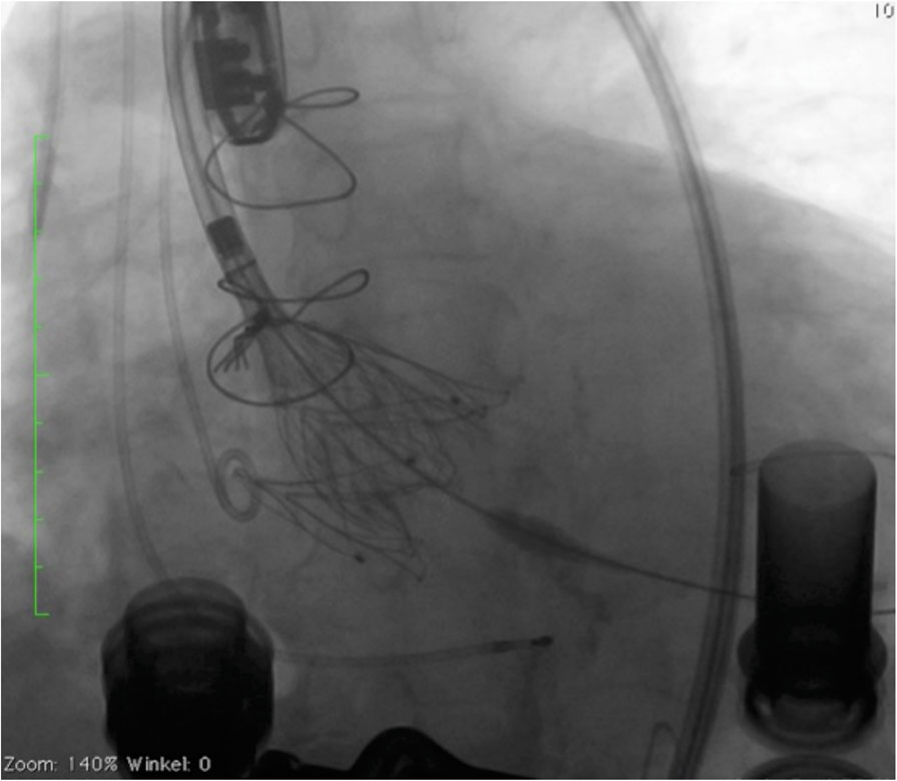
Fluoroscopy during implantation of the CoreValve prosthesis within the JenaValve prosthesis demonstrating the applied position with the distal end being positioned between the “cusp feelers” and the distal end of the JenaValve

**Fig. 5:**
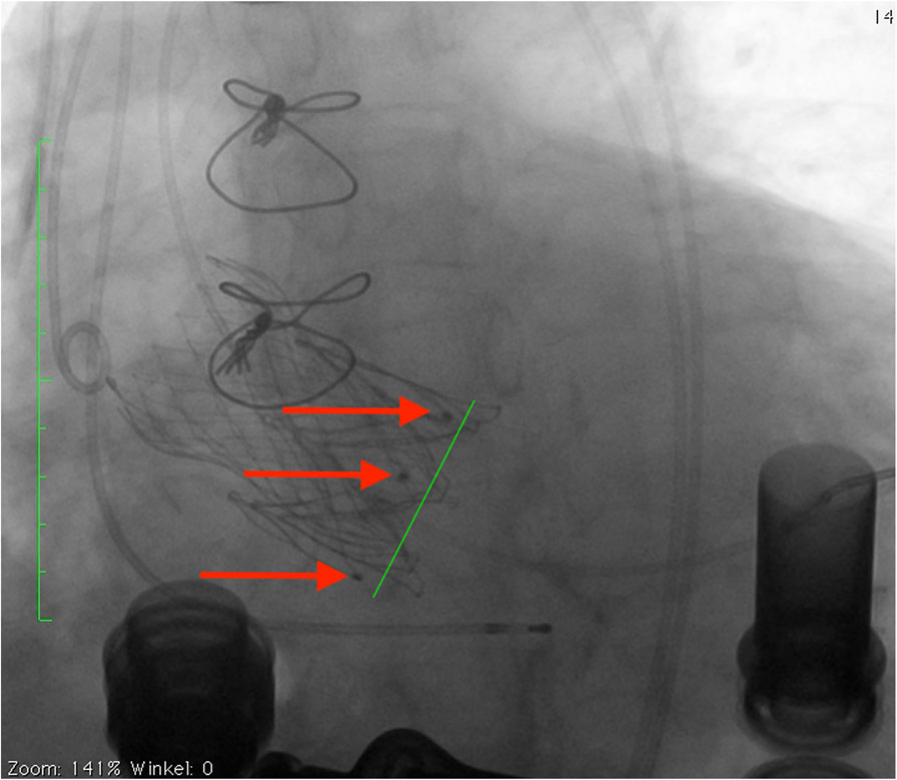
Angiogram after implantation of the CoreValve Evolut R prosthesis within the JenaValve prosthesis demonstrating planned position of the CoreValve Evolut R prosthesis (*red marker* and *green line* as planned in Fig. 1)

The procedure was performed under general anesthesia using a CoreValve Evolut R 29 mm prosthesis. The prosthesis was implanted without prior valvuloplasty. The flow rate of LVAD was reduced to minimum and pacing with a frequency of 140 beats/minute was applied during placement of the valve prosthesis. Positioning was done with great care using fluoroscopic and transesophageal echocardiography (TEE) guidance with the aim of having the ventricular strut end of the CoreValve Evolut R prosthesis between the ventricular end and the “cusp feelers” of the JenaValve prosthesis (Fig. 1). This position was obtained because of JenaValve structure and individual computed tomography analysis of our patient which had shown the ventricular edge of the JenaValve well positioned in left ventricular outflow tract (LVOT; Fig. 2). The first positioning was successful with no need for repositioning (Figs. 3 and 4). After the last fluoroscopic control the CoreValve Evolut R was released successfully in the planned position (Fig. 5). Slow rapid pacing was stopped and the LVAD flow was increased and required good hemodynamic under normal LVAD flow. His postoperative course was uneventful and he has shown a very good recovery. A second TEE did not show any change regarding the performance of the valve-in-valve and only marginal residual insufficiency. At 12-month follow-up our patient had no complaints and had a satisfactory capacity in daily life. Echocardiography showed no relevant aortic regurgitation and an increase of LVEF to 33%. At that time the 6-minute walk test was significantly increased to 381 m (compared to 148 m on admission).

## Discussion

In this report, a patient with previous TAVI and LVAD implantation developed a severe aortic insufficiency of his valve prosthesis 16 months after TAVI and 15 months after LVAD implantation. This is the first report of the implantation of a Medtronic CoreValve Evolut R heart valve prosthesis for treatment of the central severe regurgitation of a JenaValve. We discussed other transcatheter prostheses and finally decided to implant a CoreValve Evolut R prosthesis with the possibility of repositioning. The left ventricle (LV)-Edge of the CoreValve Evolut R prosthesis had to be placed slightly above the LV-Edge of JenaValve in order to adjust for the different valve cage shapes of JenaValve and the CoreValve Evolut R prosthesis and to achieve a correct valve-in-valve position and prevent subsequent “inter-prostheses” leakage. Positioning could be done using fluoroscopic guidance. The application of cardiac pacing at a rate of 140 beats/minute during the implantation of the new percutaneous heart valve resulted in a stable positioning environment and subsequent optimal placement at the desired location with only minor remaining gradient and no regurgitation.

## Conclusion

Thus, use of a CoreValve Evolut R prosthesis implanted in the described position is a valid treatment option for a transvalvularly failing JenaValve.
